# Harnessing the power of behavioural science to improve health

**DOI:** 10.2471/BLT.21.287375

**Published:** 2021-11-01

**Authors:** Elena Altieri, John Grove, Olivia Lawe Davies, Katrine Bach Habersaat, Joseph Okeibunor, Dalia Samhouri, Supriya Bezbaruah

**Affiliations:** a Department of Communications, World Health Organization, Avenue Appia 20, 1211 Geneva 27, Switzerland.; b Department of Quality Assurance, Norms and Standards, World Health Organization, Geneva, Switzerland.; c World Health Organization Regional Office for the Western Pacific, Manila, Philippines.; d World Health Organization Regional Office for Europe, Copenhagen, Denmark.; e World Health Organization Regional Office for Africa, Brazzaville, Congo.; f World Health Organization Regional Office for the Eastern Mediterranean, Cairo, Egypt.; g World Health Organization Regional Office for South-East Asia, New Delhi, India.

The international health community has set ambitious goals for health: eliminating or reducing communicable and noncommunicable diseases, ensuring appropriate use of antibiotics, increasing the uptake of vaccines and other preventive measures, and responding appropriately to epidemics, among others. Behavioural science can help in achieving these goals;^1^ more systematic use of behavioural science is needed to accelerate progress towards the sustainable development goals.^2^ The World Health Organization (WHO), as part of its transformation,^3^ is scaling up the use of behavioural science to support its Member States in achieving better health and well-being for their populations.^4^

The global health community should adopt multidisciplinary approaches to changing behaviours and integrating behavioural and social science into public health. Behavioural science is one of several disciplines that can help us understand and address behaviours that affect health – together with social sciences, anthropology, social psychology, behavioural economics, systems thinking, social marketing, human-centred design and implementation science. Each one of these disciplines is uniquely equipped to contribute to this understanding – and action – while sharing the goal of developing and implementing policies and programmes designed around people’s needs and life experiences.

This theme issue of the *Bulletin of the World Health Organization* focuses on behavioural science and its application to understand individual and community behaviours through the analysis of cognitive, social and environmental drivers and barriers. Traditionally, public health efforts addressing risk behaviours have often focused on knowledge and awareness. Behavioural science analyses influencing factors within the environments around individuals and how insights can be used to design and make discrete changes in the environment to impact behaviours.^5^

The systematic use of behavioural science to address longstanding and emerging public health challenges can provide an opportunity to improve the impact of programmes and policies, but behavioural science is still underutilized in public health. Ineffective behaviour change techniques continue to be used, while effective ones are not used or are difficult to replicate because both practitioners and researchers often do not capture, describe or understand their mechanisms of action.^6^

Such underutilization may also arise because systematically adopting behavioural science can be challenging for practitioners and policy-makers.^7^ For example, behavioural drivers and barriers can be highly specific to context, requiring continuous data collection, evaluation^5^ and tailoring to the local context. Several articles in this theme issue illustrate how not all behaviours are the same;^8^^,^^9^ how understanding and clearly defining behaviours is essential to defining the desired outcome of an intervention; and how different behavioural challenges warrant different theoretical approaches, methods and techniques, and the capacity among practitioners to distinguish among them. For example, some behaviours happen at the microlevel within a defined environment,^10^ others take place in bundles,^11^ while others involve complex chains of decisions and events that span time and space. These behaviours are influenced by doctors, nurses, public health officials, donors, nongovernmental organizations and the private sector that create socioecological systems around individuals that influence their decision-making and practices related to health.^12^ Addressing these and other challenges requires more research, investment, capacity-building, experimentation and improved collaboration between public health experts and behavioural scientists.

This theme issue of the *Bulletin* explores some of these practical and theoretical challenges with the objective of advancing the use of behavioural science within public health. We hope to do so by raising the profile of the debate on these issues and including public health experts in this discussion. Only when these practical and theoretical challenges get beyond discussions held exclusively or mostly among behavioural and social scientists will there be meaningful progress towards the systematic inclusion of behavioural science into public health.

This theme issue targets the global community of public health experts, providing examples of the benefits of incorporating behavioural science in our work. The articles show how behavioural science has been used successfully around the world in support of programmes addressing a variety of risk behaviours and health issues, from iron deficiency^13^ to trachoma,^14^ vaccine hesitancy,^15^ family planning,^16^ malaria,^17^ human immunodeficiency virus^18^ and the coronavirus disease 2019.^19^ This theme issue is part of WHO’s efforts to scale up the use of behavioural science in its work in improving the health of billions of people.
